# Retrospective observational study of interventional radiology and critical care coagulopathy

**DOI:** 10.1186/cc13284

**Published:** 2014-03-17

**Authors:** SP Hibbs, S McKechnie, M Little, M Desborough

**Affiliations:** 1 John Radcliffe Hospital, Oxford, UK

## Introduction

Estimation of bleeding risk in critical care patients undergoing interventional radiological procedures is often made on the basis of coagulation tests. If these tests are abnormal, fresh frozen plasma (FFP) is often given to reduce the risk of bleeding, despite a poor evidence base for this practice [1]. There is a relatively better evidence base for prophylactic platelet transfusion [2] but clinical practice is inconsistent. Through a retrospective study we aimed to establish the thresholds triggering use of FfP and platelet transfusion prior to percutaneous drain insertion in critical care patients.

## Methods

We identified 68 consecutive chest, abdominal or pelvic drain insertions in 54 critical care patients between 1 January 2008 and 11 October 2012 at the John Radcliffe Hospital, Oxford. The prothrombin time (PT), activated partial thromboplastin time (APTT) and platelet counts prior to each procedure were recorded to demonstrate triggers used for FFP and platelet transfusion. In patients who underwent transfusion, the next PT, APTT and platelet count post transfusion were recorded.

## Results

Patients who received FFP had a mean PT of 18.5 seconds while those who did not receive FFP had a mean PT of 16.7 seconds (unpaired *t *test, *P *= 0.275). In the nine patients given FFP, the pretransfusion mean PT was 18.5 seconds whereas the post-transfusion mean PT was 17.1 seconds (paired *t *test, *P *= 0.235). The pre-transfusion mean APTT was 41.6 seconds compared with a post-transfusion mean APTT of 38.1 seconds (paired *t *test, *P *= 0.127). No patient had platelet levels below the recommended transfusion threshold [2], but one patient nevertheless received a double-dose platelet transfusion. One patient had a recorded immediate bleeding complication. Their PT was 15.6 seconds and APTT was 40.9 seconds and they did not receive FFP. One patient had an anaphylactic reaction whilst receiving FFP.

## Conclusion

This study demonstrates inconsistent use of FFP, with no significant difference in PT between patients who were transfused and those that were not. The lack of effect of FFP transfusion on PT and APTT creates additional confusion for its prophylactic usage. There is a need for further clarification around coagulopathy and interventional radiology in the critical care setting. The low absolute incidence of bleeding complications and risk of complications from transfusion lends further support to the view that FFP should be used therapeutically rather than as prophylactic 'cover' [1].
